# Resource utilization associated with extracorporeal membrane oxygenation vs. microaxial flow pump for infarct-related cardiogenic shock

**DOI:** 10.1093/ehjacc/zuaf024

**Published:** 2025-02-12

**Authors:** Margriet Bogerd, Luc ten Hoorn, Sanne ten Berg, Elma J Peters, Annemarie E Engström, Arjan Malekzadeh, Holger Thiele, Jacob E Møller, Christian Hassager, Alexander P J Vlaar, José P S Henriques

**Affiliations:** Department of Cardiology, Amsterdam UMC, Amsterdam, The Netherlands; Department of Cardiology, Amsterdam UMC, Amsterdam, The Netherlands; Department of Cardiology, Amsterdam UMC, Amsterdam, The Netherlands; Department of Cardiology, Amsterdam UMC, Amsterdam, The Netherlands; Department of Intensive Care Medicine, Amsterdam UMC, Amsterdam, The Netherlands; Medical Library, Amsterdam UMC, Amsterdam, The Netherlands; Department of Internal Medicine/Cardiology, Heart Center Leipzig at Leipzig University and Leipzig Heart Science, Leipzig, Germany; Department of Cardiology, Copenhagen University Hospital—Rigshospitalet, Copenhagen, Denmark; Department of Cardiology, Odense University Hospital, Odense, Denmark; Department of Cardiology, Copenhagen University Hospital—Rigshospitalet, Copenhagen, Denmark; Department of Intensive Care Medicine, Amsterdam UMC, Amsterdam, The Netherlands; Department of Cardiology, Amsterdam UMC, Amsterdam, The Netherlands

**Keywords:** Myocardial infarction, Cardiogenic shock, Microaxial flow pump, Extracorporeal membrane oxygenation, Costs, Resource utilization

## Abstract

**Aims:**

Microaxial flow pump and venoarterial extracorporeal membrane oxygenation (VA-ECMO) are increasingly used in infarct-related cardiogenic shock. This study provides a comparative overview of real-world resource utilization associated with these devices (PROSPERO: CRD42024505174).

**Methods and results:**

EMBASE, MEDLINE, and Cochrane Library were sought from inception to 13 November 2024 for studies reporting at least one primary outcome, including intensive care unit (ICU) length of stay (LOS), hospital LOS, in-hospital costs, and discharge destination. In-hospital mortality was included as secondary outcome. This study was guided by the PRISMA-2020 guideline. Study selection and data extraction were independently performed by two researchers. Risk-of-bias assessments were done using the Newcastle-Ottawa-Scale. Data were pooled using random-effect models. In total, 12 retrospective cohorts were identified encompassing 92 262 microaxial flow pump- and 16 474 VA-ECMO patients data. The meta-analysis of hospital LOS and in-hospital costs revealed favourable results for the microaxial flow pump, with mean differences (MD) of −5.3 days (95% CI: −6.6, −4.1) and −$113 983 (95% CI: −$143 153, −$84 812), respectively. Microaxial flow pump survivors were also 45% more likely to be discharged home (95% CI: 1.28–1.64). Intensive care unit-length of stay was reported by one study, reporting a 10 days MD in favour of the microaxial flow pump. The averaged in-hospital mortality rates were 44% and 57% for the microaxial flow pump and VA-ECMO, respectively. An inherent limitation of observational studies is confounding by indication.

**Conclusion:**

Microaxial flow pump was associated with lower resource utilization compared with VA-ECMO. Resource utilization should be incorporated in prospective RCTs and taken into account when considering these devices.

## Graphical Abstract

**Graphical Abstract:**
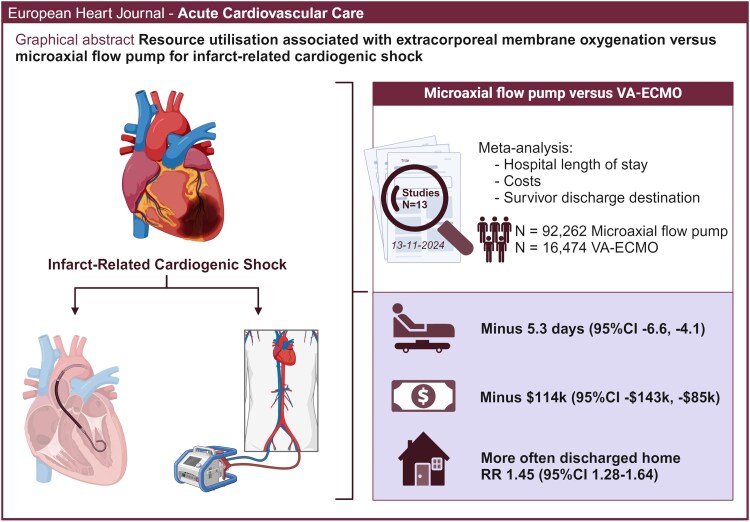
Created in BioRender. Henriques, J. (2025) https://BioRender.com/f12d296

## Introduction

Cardiogenic shock (CS) occurs in 3–10% of the acute myocardial infarctions (AMI) and causes end-organ hypoperfusion, end-organ failure, and even death in 40–50% of the cases.^1–5^ In these patients, mechanical circulatory support (MCS) may be considered according to the European and American guidelines, albeit with limited guidance on device choice.^6,7^ Around 20–25% of the acute myocardial infarction–related cardiogenic shock (AMICS) patients in Europe and up to 40% in the USA currently receive MCS.^2,8–10^ The usage of the microaxial flow pump and venoarterial extracorporeal membrane oxygenation (VA-ECMO) has increased rapidly over the last few years, despite the lack of prospectively established beneficial evidence at the time.^8,11–15^ While randomized trials evaluating VA-ECMO in AMICS fail to show better clinical outcome, the DanGer-Shock trial demonstrated that the microaxial flow pump was associated with a reduction in 180-day mortality in a highly selected, non-comatose, ST-elevation myocardial infarction (STEMI) population.^13,15,16^ Whether this effect is related the microaxial flow pump as support device (in contrast to VA-ECMO) or related to adequate patient selection remains a matter of debate. Namely, the recent individual patient data (IPD) meta-analysis of all randomized trials evaluating MCS in AMICS showed a 180-day mortality reduction of MCS (including both microaxial flow pump and VA-ECMO) in highly selected, non-comatose STEMI patients. Importantly, this is at the cost of increased incidences in major bleeding and vascular complications.^17^

Previous systematic reviews have indicated that microaxial flow pump is associated with lower adverse event rates compared with VA-ECMO.^18–20^ Although factors like adverse event occurrence influence resource utilization, a comparative overview on this topic is lacking, eventhough both devices are highly resource-intensive. Given the current trend of rising healthcare expenditure, together with the rising usage of MCS devices, resource utilization might be considered when opting for one of these devices.^21–23^ The aim of this review was to give insight into and compare resource utilization associated with the use of the microaxial flow pump vs. VA-ECMO in AMICS patients. To this end, four primary outcomes reflecting resource utilization were evaluated, including intensive care unit (ICU) length of stay (LOS), hospital LOS, in-hospital costs and discharge destination reflecting post-discharge resource utilization.

## Methods

### Data source and search strategy

This systematic review with meta-analysis was guided by the Preferred Reporting Items for Systematic Reviews and Meta-Analyses (PRISMA) 2020 statement and has been registered in the PROSPERO (International Prospective Register of Systematic Reviews) registry under the identification number CRD42024505174. The search strategy followed an iterative approach. First, preliminary exploratory searches were performed by two researches (L.t.H. and M.B.), identifying relevant keywords and studies. These were discussed and a final query was formulated together with a librarian (A.M.). The comprehensive online search of the electronic databases MEDLINE, EMBASE and the Cochrane Library was performed to identify all studies from inception up to 13–11-2024 comparing microaxial flow pump and VA-ECMO in the setting of AMICS (Graphical Abstract). Key search terms included ‘acute myocardial infarction’, ‘acute coronary syndrome’, ‘cardiogenic shock’, ‘mechanical circulatory support’, ‘percutaneous ventricular assist device’, ‘Impella’, ‘microaxial flow pump’ and ‘extracorporeal membrane oxygenation’. The full search strategies can be found in the supplements, Supplementary material online, *Table S1A–C*.

### Study selection

All unique records were uploaded in Rayyan and independently screened by two out of three researchers (M.B., S.t.B., L.t.H.), after the removal of duplicates. Studies were first screened on title and abstract, the remaining studies were screened based on full text. Disagreements regarding inclusion and exclusion among the researchers were resolved by the third researcher. Eligible studies (both prospective and retrospective) were included if they described adults (>18 years old) with CS caused by AMI (in at least 50% of the included subjects) and compared the usage of microaxial flow pump or percutaneous ventricular assist device (pVAD) with VA-ECMO. Studies had to report at least one of the following primary outcomes: ICU-LOS, hospital LOS, in-hospital costs, and/or discharge destination. The secondary outcome ‘in-hospital mortality’ was not required. Studies comparing the usage of VA-ECMO with the usage of VA-ECMO plus unloading were excluded. Animal studies, abstracts only, case reports or case series (defined as cohort of <10 patients per treatment arm), systematic reviews and meta-analyses were excluded. Cross-referencing was performed to check for additional relevant studies. A PRISMA flow diagram was made to visualize the screening process.

### Data extraction and quality assessment

Data were extracted from the included studies independently by two researchers (M.B. and L.t.H.) through a pre-specified case report form (see Supplementary material online, *Table S2*). Data extraction included study specific data (authors, title, year of publication, country, data type and source, time of data query, total number of patients, percentage of patients with AMI and the number of patients in the microaxial flow pump and VA-ECMO cohort), cohort-specific data [age, percentage of males, percentage of patients with diabetes mellitus, percentage of patients that suffered an out-of-hospital cardiac arrest (OHCA), the admission level of lactate reported, the percentage of patients that were treated with a percutaneous coronary intervention (PCI) and with coronary artery bypass grafting (CABG) and the percentage of patients with concomitant usage of intra-aortic balloon pump (IABP)], resource utilization data (ICU-LOS, hospital LOS, reported in-hospital costs, and the percentage of survivors discharged home) and outcome data (in-hospital mortality). If data were not directly available [e.g. reported for multiple groups (e.g. male/female)], the combined mean and standard deviation or incidences were calculated according to the Cochrane Handbook for Systematic Reviews of Interventions.^24^ The bias assessment was also performed independently by two researchers (M.B. and L.t.H.), according to the Newcastle-Ottawa-scale (NOS).^25^ Disagreements in data extraction and risk of bias assessment were resolved through discussion with a third author (S.t.B.). Extracted data and risk-of-bias assessment were visualized using tables.

### Statistical analyses

The meta-analysis was performed using the Review Manager 5.4.0 software. Dichotomous data (in-hospital mortality and discharge destination) were pooled using the Mantel-Haenszel random effects model to calculate the weighted risk ratio (RR) with 95% confidence interval (CI). Continuous data (LOS and in-hospital costs) were pooled using inverse variance random effects model to calculate the standardized mean difference (SMD) and mean difference (MD) with 95% CI for in-hospital costs and LOS, respectively. In order to incorporate all available data into the meta-analysis, we assumed normality for data reported as median (IQR), resulting in mean equals median and SD equals IQR/1.35.^24^ Meta-analysis plots were made to visualize the separate and pooled study effects. Heterogeneity was calculated using the I^2^-statistics, with percentages of <25%, 25–75%, and >75% indicating low, intermediate, and high heterogeneity, respectively. To assess the impact of database weight inflation due to double-counting, study overlap based on data origin and timeframe was identified, and sensitivity analyses were performed retaining only the most comprehensive studies. In addition, sensitivity analyses were used to assess the impact of outliers, intercontinental differences, and the robustness of the results under various inclusion and exclusion criteria and different statistical assumptions. Significant outliers were not incorporated in the primary results. Funnel plots based on fixed-effect models were used to visually assess publication bias. To prevent Type I error due to four primary outcomes, the Bonferroni correction was applied, resulting in a two-tailed alpha of 0.013 for the primary outcomes. Secondary- and additional analyses are to be interpreted as hypothesis-generating.

## Results

**Figure 1:**
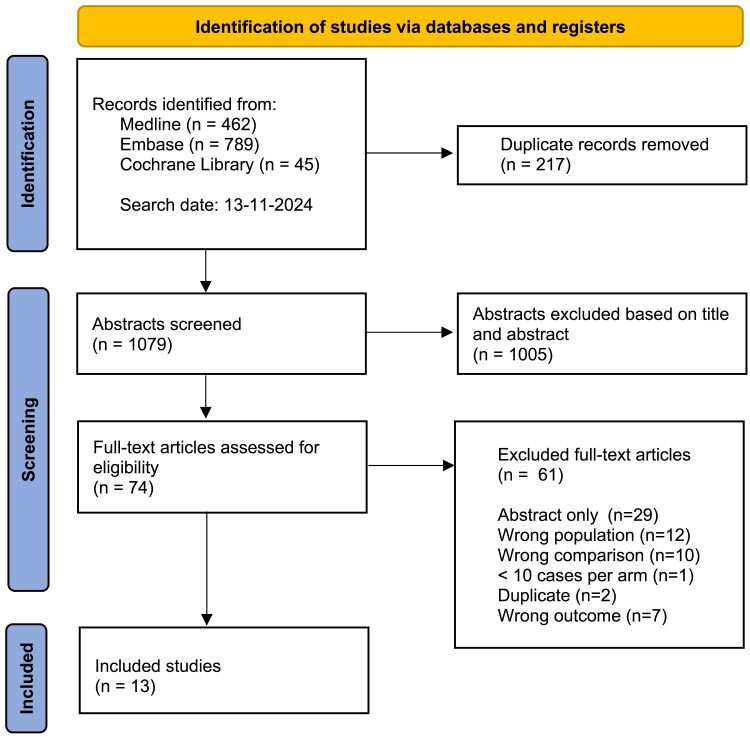
PRISMA flowchart.

The systematic search identified 1079 unique records, of which 1005 were excluded based on title or abstract. Full-text screening of the remaining studies resulted in 13 studies included for further analysis (*Table 1*, Graphical Abstract).^2,26–36^  *Figure 1* presents a PRISMA flow diagram outlining the reasons for study exclusion (see *Figure 1*. PRISMA flowchart).

**Table 1:** Study and cohort characteristics

First author (year of publication)	Country (time)	Data type	AMICS, *n* (%)	Device	*n*	Age (years, ±SD/(IQR))	Male (%)	DM (%)	OHCA (%)	Lactate (mmol/L)	PCI (%)	CABG (%)	IABP (%)
Maini (2014)	USA (2009–2011)	Retrospective; claims database (medPAR)	1188 (100)	MFP	883	69.2	65	17	—	—	—	—	—
				ECMO	305	63.8	67	17	—	—	—	—	—
Karami (2020)	Netherlands (2006–2018)	Retrospective; chart review	128 (100)	MFP	90	60 ± 10	73	17	50	6.3 ± 4.0	100	0	4
				ECMO	38	55 ± 9	79	11	24	7.1 ± 4.8	100	0	55
Lemor (2020)	USA (2015–2017)	Retrospective; claims database (HCUP-NIS)	6290 (100)	MFP	5730	65.6 ± 11.8	73	40	—	—	100	3	17
				ECMO	560	61.2 ± 11.6	83	41	—	—	100	11	52
Vallabhajosyula (2020)	USA (2005–2016)	Retrospective; claims database (HCUP-NIS)	374 920 (100)	MFP	7516	65.1 ± 12.1	71	—	35	—	77	—	0
				ECMO	1469	58.1 ± 11.1	73	—	43	—	25	—	0
Pahuja (1) (2021)	USA (2005–2014)	Retrospective; claims database (HCUP-NIS)	172 491 (100)	MFP	2079	—	—	—	—	—	—	—	0
				ECMO	444	—	—	—	—	—	—	—	0
Pahuja (2) (2021)	USA (2005–2014)	Retrospective; claims database (HCUP-NIS)	172 491 (100)	MFP	2078	—	—	—	—	—	—	—	0
				ECMO	444	—	—	—	—	—	—	—	0
Vetrovec (2021)	USA (2015–2017)	Retrospective, PS-matched,(Medicare FFS)	2850 (100)	MFP	338	—	70	42	—	—	—	—	—
				ECMO	338	—	71	39	—	—	—	—	—
Vojjini (2021)	USA (2012–2017)	Retrospective; claims database (HCUP-NIS)	90 071 (100)	MFP	9890	65.2 ± 12.0	72	—	34	—	78	—	0
				ECMO	1386	58.9 ± 11.5	75	—	43	—	31	—	0
Bogerd (2023)	Germany (2020–2021)	Retrospective; claims database (InEK)	4088 (100)	MFP	2700	-	75	31	18	—	100	—	1
				ECMO	959	-	79	24	40	—	100	—	5
Briasoulis (2023)	USA (2016–2019)	Retrospective; claims database (HCUP, NRD)	80 997 (100)	MFP	9055	64.6 ± 12.1	73	38	—	—	-	—	0
				ECMO	753	57.4 ± 12.0	76	35	—	—	—	—	0
Padberg (2024)	Germany (2010–2017)	Retrospective; claims database (AOK)	39 864 (100)	MFP	776	70.6(17.3)	70	52	0	—	95	2	—
				ECMO	833	63.7(16.9)	77	51	0	—	79	27	—
Buda (2024)	USA (2016–2020)	Retrospective; claims database (HCUP,NRD)	294 839 (100)	MFP	33577	66.5 ± 12.0	72	44	—	—	71	9	—
				ECMO	8067	57.5 ± 15.2	70	32	—	—	27	21	—
Ali (2024)	USA (2016–2020)	Retrospective; claims database (HCUP, NRD)	20 950 (100)	MFP	19628	68 (17)	71	46	—	—	100	0	—
				ECMO	1322	61 (15)	72	38	—	—	100	0	—

MFP, microaxial flow pump; ECMO, venoarterial extracorporeal membrane oxygenation; medPAR, Medicare Provider Analysis and Review; HCUP, Healthcare Cost and Utilisation Project; NIS, National Inpatient Sample; FFS, Fee-For-Service; InEK, Institute for the Hospital Remuneration System; NRD, National Readmission Database; AOK, ‘Allgemeine Ortskrankenkasse’ or general regional health insurance.

### Qualitative assessment and study characteristics

A total of 12 retrospective cohorts were identified among the 13 included studies, reporting on data from AMICS patients who received microaxial flow pump support (*n* = 92 262) or VA-ECMO (*n* = 16 474; Graphical Abstract). The two studies of Pahuja *et al*.^30,31^ described the exact same cohort. All but one study were published in 2020 or after, inclusion periods varied between 2005 and 2021. The study from Karami *et al*.^27^ was based on a Dutch chart review study. Bogerd *et al*.^2^ and Padberg *et al*.^35^ reported on German claims data and Vetrovec reported USA claim-based propensity matched data.^32^ The majority of studies were based on USA claims data, including some overlapping timeframes.^30,31^ All studies yielded an intermediate risk of bias (see Supplementary material online, *Table S3*). For cost-estimation, the majority of the studies used admission charges [International Classification of Disease (ICD) and Diagnosis-Related-Group (DRG) based], which were converted into costs using validated Cost-to-Charge Ratios (CCR).^26,28–31,33,34,36,37^ Vetrovec *et al*.^32^ described Medicare Fee-For-Service (FFS) data, thereby analysing the actual provider incurred costs, including the device costs. Bogerd *et al*.^2^ used a combination of admission costs (DRG based) and additional procedural fees (including device-costs). Overall, the studies described a majority of male patients (±73%) between 55 and 71 years old. Microaxial flow pump-supported patients were considerably older in all age-reporting studies. The incidence of OHCA was reported in five studies and varied between 0 and 50%.^2,27,29,33,35^ Three out of these five studies reported a higher incidence of OHCA in the VA-ECMO cohort.^2,29,33^ With regards to revascularization (PCI or CABG) strategy, in only four studies all patients underwent PCI.^2,27,28,37^ In four studies, the percentages of patients that underwent PCI were significantly lower among the VA-ECMO cohort compared with the microaxial flow pump cohort.^29,33,35,36^ The percentage of patients that underwent CABG in the VA-ECMO cohort was reported higher by three studies.^28,35,36^  *Table 1* shows an elaborate overview of the included studies. Four studies reported data for patients that were treated with two or more devices (including IABP). The patient characteristics and outcomes of these patients are presented in Supplementary material online, *Table S4*.

### Meta-analysis of the primary outcomes

*Table 2* summarizes the primary and secondary outcomes reported per included study. The study of Briasoulis *et al*. was identified as a distorting outlier and was therefore excluded from the primary meta-analysis. The rationale for initial exclusion and the meta-analyses including this study are presented in the Supplementary material online, *Figure S5A–D*.

**Table 2:** Outcomes per study

Study characteristics			Primary outcomes				Secondary Outcome
First author (year of publication)	Device	*n*	ICU LOS (days)	Hospital LOS (days)	In-hospital costs	% of survivors discharged home	In-hospital mortality (%)
Maini (2014)	MFP	883	—	13.2	$90 929	—	44
	ECMO	305	—	17.9	$144 257	—	58
Karami (2020)	MFP	90	6 (IQR 3–14)	—	—	—	53
	ECMO	38	16 (IQR 9–30)	—	—	—	50
Lemor (2020)	MFP	5730	—	7	$66 078	52	41
	ECMO	560	—	11	$122 996	31	46
Vallabhajosyula (2020)	MFP	7516	—	11.2 ± 12.8	$314 000 ± $259 000	52	44
	ECMO	1469	—	18.4 ± 23.7	$566 000 ± $589 000	42	61
Pahuja (1) (2021)	MFP	2079	—	12^a^	$306 454	—	41
	ECMO	444	—	26^a^	$507 657	—	56
Pahuja (2) (2021)	MFP	2078	—	15^a^	$305 480	—	41
	ECMO	444	—	26^a^	$484 148	—	56
Vetrovec (2021)	MFP	338	—	12.12	$101 328	—	52
	ECMO	338	—	16.59	$153 723	—	64
Vojjini (2021)	MFP	9890	—	9.6 ± 10.9	$323 780 ± $246 922	50	43
	ECMO	1386	—	18.9 ± 25.3	$602 312 ± $678 738	35	58
Bogerd (2023)	MFP	2700	—	11.4 ± 16.4	€36 655	52	61
	ECMO	959	—	13.8 ± 19.2	€43 323	33	67
Briasoulis (2023)	MFP	9055	—	15.9 ± 26.7	$139 567 ± $140 785	54	59
	ECMO	753	—	8.8 ± 9.0	$50 884 ± $40 575	73	29
Padberg (2024)	MFP	776	—	6 (IQR 22)	—	—	67
	ECMO	833	—	12 (IQR 30)	—	—	74
Buda (2024)	MFP	33 577	—	9 (IQR 3–17)	$81 808 [$55 372-$131 217]	—	44
	ECMO	8067	—	14 (IQR 6–30)	$157 674 [$92 726-$268 671]	—	54
Ali (2024)	MFP	19 628	—	7 (IQR 12)	$89 383 (IQR $66 582)	—	44
	ECMO	1322	—	11 (IQR 19)	$144 587 (IQR $144 982)	—	52

ICU, intensive care unit; LOS, length of stay; MFP, microaxial flow pump; ECMO, venoarterial extracorporeal membrane oxygenation; IQR, interquartile range.

^a^Length of stay of survivors only, not incorporated in the meta-analysis.

**Figure 2:**
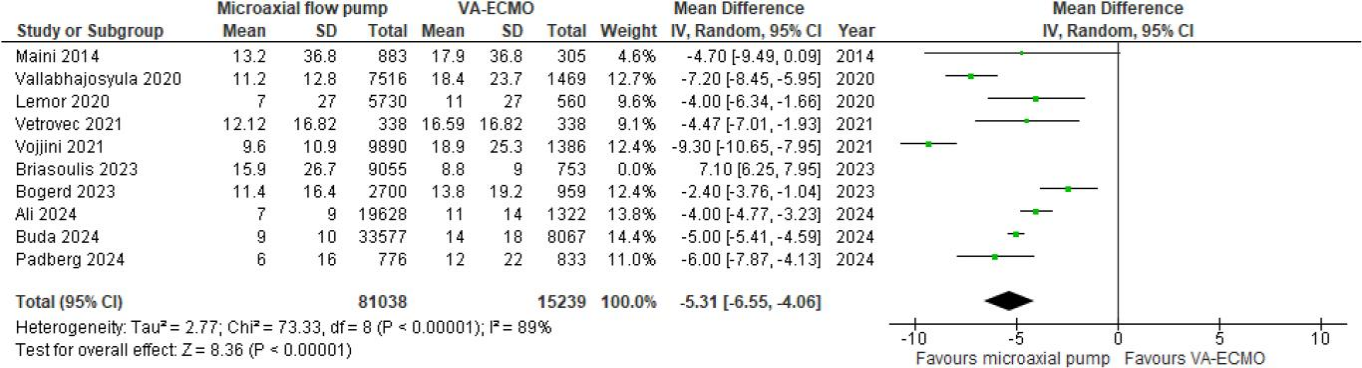
Meta-analysis of hospital length of hospital stay.

**Figure 3:**
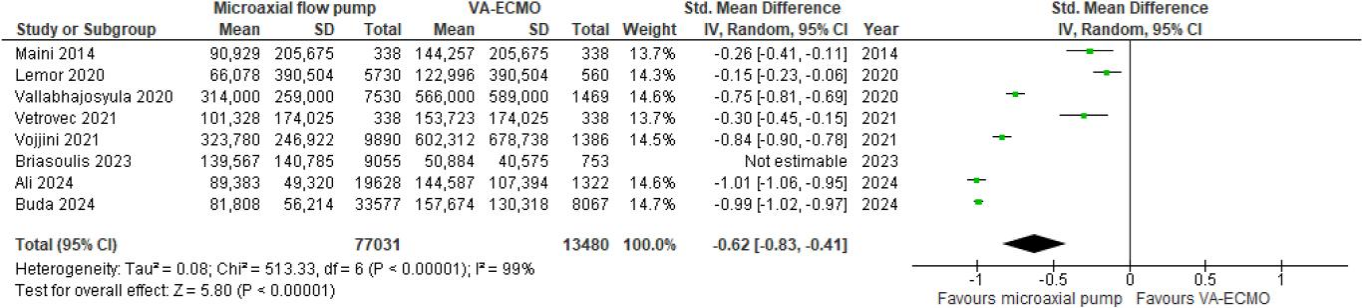
Meta-analysis of in-hospital costs.

**Figure 4:**
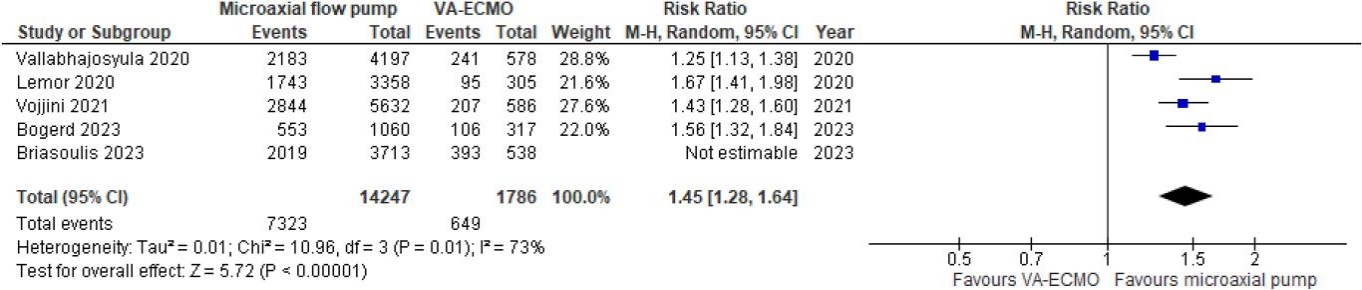
Meta-analysis of discharge destination.

ICU-LOS was reported by only one study, showing a mean difference (MD) of 10 days in favour of the microaxial flow pump (*Table 2*).^27^ The averaged hospital LOS was significantly shorter in microaxial flow pump vs. VA-ECMO patients, with a MD of −5.31 (95% CI −6.55, −4.06, *P* < 0.001, I^2^ = 89%; *Figure 2*). Similarly, the averaged in-hospital costs associated with the microaxial flow pump were significantly lower compared with VA-ECMO, with a MD of −$113 983 (95% CI: −$143,153, −$84,812, *P* < 0.001, I^2^ = 98%) and a corresponding standardized mean difference (SMD) of −0.62 (95% CI: −0.83, −0.41, *P* < 0.001, I^2^ = 99%; *Figure 3*). In addition, 51% in the microaxial flow pump survivors and 36% of the VA-ECMO survivors were discharged home, with a corresponding RR of 1.45 (95% CI: 1.28, 1.64, *P* < 0.001, I^2^ = 73%; *Figure 4*). The averaged in-hospital mortality rates associated with the microaxial flow pump and VA-ECMO were 44% and 57% respectively (see Supplementary material online, *Figure S6*).

### Sensitivity analysis

All primary and secondary outcomes showed similar patterns between the devices after exclusion of overlapping cohorts (Supplementary material online, *Figure S7A–D*). No important differences where identified when comparing studies from Europe and the USA (Supplementary material online, *Figure S8A–F*), and findings remained consistent in studies exclusively involving 100% PCI. (see Supplementary material online, *Figure S9A*–*D*).^2,27,28^ The meta-analysis using fixed-effect models and the corresponding funnel plots are presented in Supplementary material online, *Figure S10A*–*D*.

## Discussion

This review and meta-analysis is the first to provide a comprehensive overview and comparison of the resource utilization associated with the usage of the microaxial flow pump vs. VA-ECMO in the setting of AMICS. The results indicate that in a real-world setting, haemodynamic support with the microaxial flow pump is associated with lower resource utilization compared with VA-ECMO. More specifically, the microaxial flow pump was associated with shorter hospital LOS (MD 5.3 days), lower in-hospital costs (MD: $113 983), and more survivors being discharged home directly (RR: 1.45, 95% CI: 1.28–1.64; Graphical Abstract). Intensive care unit-length of stay was reported by one study only (MD: 10 days in favour of microaxial flow pump). In addition, microaxial flow pump supported patients yielded lower in-hospital mortality.

Over the last few years, a remarkable surge in the usage of the microaxial flow pump and VA-ECMO in AMICS has been observed.^8,11,12^ This surge has occurred despite the absence of high-quality evidence demonstrating benefit of these devices. In fact, two randomized trials on VA-ECMO, the ECMO-CS trial (*n* = 122) and the ECLS-SHOCK (*n* = 420) trial, showed no outcome improvements associated with VA-ECMO.^13,15^ More recently, the long-awaited DanGer-Shock trial (*n* = 360) was published, demonstrating a survival benefit (*P* = 0.04) of the microaxial flow pump compared with standard of care alone in highly selected non-comatose STEMI only patients.^16^ Importantly, the designs of the aforementioned trials differed significantly (e.g. in- and exclusion criteria) and inter-trial or inter-device comparisons therefore remain challenging. Actually, the more recently published IPD meta-analysis that combined all randomized data on MCS in AMICS showed that, in highly selected STEMI patients without hypoxic brain injury, overall MCS use (including both VA-ECMO and microaxial flow pump) reduced 6 month mortality compared with control.^17,38^ A randomized head-to-head comparison is still lacking.

Given the ongoing absence of consensus regarding the additive values of the devices on clinical outcome, associated resource utilization might be of importance when considering one of these devices in clinical practice. This is particularly important given their increasing use and the globally rising proportion of health expenditure relative to the gross domestic product (GDP).^8,11,12,23^

Although confounding by indication is an inherent limitation of observational studies, the observed resource utilization does resemble real-world practice. In addition, observed differences between the microaxial flow pump and VA-ECMO cohorts are interesting and shed additional light on the data. For instance, across all included studies, microaxial flow pump-supported patients were significantly older. Advanced age is associated with poorer outcomes across all stages of CS.^39^ Surprisingly, poorer outcomes were observed in the younger VA-ECMO patients. Another important difference was the higher percentage of OHCA in the VA-ECMO cohort, this might explain the observed differences in discharge destinations due to potential severe permanent neurological damage.^2,27,29,33,35^ However, that possibly also implicates more neurological deaths and therewith shorter LOS in the VA-ECMO cohort, which is not supported by the current findings. Another potential confounder explaining the observed difference in discharge destination might be differences in institutional characteristics between microaxial flow pump and VA-ECMO-supported patients. That is, VA-ECMO treatment might have been reserved to specialized tertiary centres only, discharging to their referral centres. In that case, the true LOS of VA-ECMO patients would then actually be longer than currently presented.

Opposite to potential confounders, the primary outcome results may also be explained by inherent device characteristics. Previous meta-analyses consistently indicate higher complication rates in VA-ECMO-supported patients.^18,19,40^ Complications likely contribute to longer ICU- and hospital LOS, increased in-hospital costs, and a smaller proportion of survivors being discharged home directly.^41^ This meta-analysis was not aimed to draw any conclusions on device differences with respect to in-hospital mortality, which has merely been included for reference as secondary outcome. For device differences upon mortality, we refer to the recently published RCTs on this topic.^13,16,17^

This review focused solely on the use of the microaxial flow pump or VA-ECMO, while concomitant usage (ECPELLA) is often deployed.^42^ RCTs investigating whether unloading is beneficial and with what device are still underway (REMAP ECMO [NCT05913622], UNLOAD ECMO [NCT05577195]). Nevertheless, a large and contemporary paper demonstrated that concomitant or consecutive use of a microaxial flow pump and VA-ECMO was associated with even higher resource utilization than VA-ECMO alone.^2^ Three other studies assessing resource utilization in patients with two or more devices did not show increased resource utilization compared with patients receiving only one device. However, these reports also included the concomitant or consecutive use of an IABP, which is unlikely to have a major effect on resource utilization in contrast to the microaxial flow pump and/or VA-ECMO.^29,33,34^

This meta-analysis shows that microaxial flow pump support was associated with less resource utilization compared with VA-ECMO, and we believe this should be considered when opting for MCS. Especially considering the globally rising proportion of healthcare expenditure relative to GDP and the at best limited additive value of VA-ECMO in particular.^13,15,23^ Nevertheless, well-designed RCTs are continuously needed to truly dictate the optimal treatment strategy in AMICS patients. For a selected patient population of STEMI patients without hypoxic brain injury, the microaxial flow pump, and potentially MCS in general, appears beneficial.^16,17,38^ The single-nation ULYSS trial (NCT053664552; *n* = 204) will have to reiterate the results of the DanGer-Shock trial, especially given that the industry-sponsored RECOVER IV trial (*n* = 560) has been stopped.^16,43^ Further prospective comparative RCTs are warranted and should incorporate resource utilization to confirm these findings.

### Limitations

This review has some inherent limitations. It was confined to retrospective studies and primarily reliant on claims data, inherently introducing confounding by indication and information availability. Nevertheless, these studies reflect actual current research utilization in real-world practice, where patient selection is an integral component. Intensive care unit-length of stay was reported by one study only, thereby limiting generalizability. Given the high heterogeneity among the included studies, random effect models were used to assess the effects while accounting for between-study differences. There was considerable overlap between studies. However, double counting did not unjustly inflate the results as demonstrated by the sensitivity analysis. Noteworthy, although length of stay is often not normally distributed, we incorporated these data into the meta-analysis. Also, a small though not precisely quantified proportion of the microaxial flow pump cohort received TandemHeart instead. Evidence however suggests that TandemHeart is associated with longer length of stay and higher in-hospital costs compared with microaxial flow pump, indicating that the results would have been even more outspoken without these TandemHeart patients.^44^ Lastly, this review was confined to the usage of microaxial flow pump or VA-ECMO only, while concomitant usage (ECPELLA) has also often been deployed in the last few years and is likely to be associated with even higher resource utilization.^2^

## Conclusion

This is the first systematic review to describe and compare resource utilization associated with the microaxial flow pump and VA-ECMO usage in AMICS patients. In this meta-analysis, the use of microaxial flow pump was associated with lower resource utilization compared with VA-ECMO usage. These findings should be considered when opting for microaxial flow pump or VA-ECMO. Prospective comparative RCTs are warranted and should incorporate resource utilization to confirm these findings.

## Supplementary Material

zuaf024_Supplementary_Data

## Data Availability

The data underlying this article are available in the article and in its online supplementary material.
