# Supplementary material for: Resource utilization associated with extracorporeal membrane oxygenation vs. microaxial flow pump for infarct-related cardiogenic shock
Source: Eur Heart J Acute Cardiovasc Care. 2025 Feb 12;14(5):279–87. doi: 10.1093/ehjacc/zuaf024 (PMC12082289; doi:10.1093/ehjacc/zuaf024)

# Supplementary Material

Supplemental Tables S1a-S1c: Search strategies in MEDLINE(1a), EMBASE(1b) and COCHRANE(1c)

Supplemental Table S2: Case Report Form

Supplemental Table S3: Risk-of-Bias assessment according to the Newcastle-Ottawa Scale

Supplemental Table S4: Data for cohort of ≥ 2 MCS devices.

Supplemental Figures S5: Primary outcomes and in-hospital mortality including Briasoulis et al.

Supplemental Figure S6: Meta-analysis of in-hospital mortality

Supplemental figures S7: Sensitivity analysis – Studies excluded to prevent overlap

Supplemental Figures S8: Sensitivity analysis –Studies from Europe vs. United States of America

Supplemental figures S9: Sensitivity analysis - Only studies with 100% PCI

Supplemental Figures S10: Fixed-effect models and funnel plots

**Supplemental Table S1a: Search in MEDLINE**

| Database(s): Ovid MEDLINE(R) ALL 1946 to November 12, 2024 | |  |
| --- | --- | --- |
| Search Strategy: | |  |
| # | Searches | Results |
| 1 | exp Myocardial Infarction/ or exp Acute Coronary Syndrome/ or exp ST Elevation Myocardial Infarction/ | 216504 |
| 2 | ((cardi* or heart or myocardial*) adj3 (attack* or infarct* or failure* or arrest*)).ti,ab,kf. | 528153 |
| 3 | ((acute adj2 coronary adj2 syndrome) or AMICS or STEMI or NSTEMI or AMI).ti,ab,kf. | 71165 |
| 4 | (Left adj2 Ventricular adj2 Dominant adj2 Refractory).ti,ab,kf. | 1 |
| 5 | or/1-4 | 602933 |
| 6 | exp Shock, Cardiogenic/ | 11547 |
| 7 | ((cardi* or heart or myocardial* or cardiovascular or cardiocirculatory or circulatory) adj3 (shock* or collapse)).ti,ab,kf. | 27593 |
| 8 | (acute adj2 (heart or cardi* or myocardial* or cardiovascular or cardiocirculatory) adj2 failure).ti,ab,kf. | 14297 |
| 9 | or/6-8 | 43956 |
| 10 | (Impella* or (percutaneous adj2 ventricular adj2 assist adj2 device) or "pLVAD" or "pVAD" or "pMCS" or (microaxial adj2 flow adj2 pump)).ti,ab,kf. | 3490 |
| 11 | ((percutaneous or temporary) adj3 (circulat* or mechanical*) adj2 support*).ti,ab,kf. | 1046 |
| 12 | (percutaneous adj2 Mechanical* adj2 circulatory adj2 support).ti,ab,kf. | 206 |
| 13 | or/10-12 | 4219 |
| 14 | exp Extracorporeal Membrane Oxygenation/ | 16817 |
| 15 | (CardioHelp or RotaFlow or ECLS or ECMO or vaECLS or vaECMO or (venoarterial adj2 extracorporeal adj2 support)).ti,ab,kf. | 16472 |
| 16 | ((extracorporeal or "extra corporeal") adj2 (oxygen* or support)).ti,ab,kf. | 23515 |
| 17 | or/14-16 | 28735 |
| 18 | 5 and 9 and 13 and 17 | 662 |
| 19 | (case report or review).pt. | 3410982 |
| 20 | case report.ti. | 347090 |
| 21 | ((exp animals/ or exp veterinary medicine/ or animal*.jw.) not exp humans/) or (experiment* model* or animal* or monkey* or sheep or ?ovine or lamb* or goat* or pig* or swine or porcine or pup* or dog* or canine or bitch* or beagle* or feline or rodent* or rabbit* or rat or rats or mouse or murine or mice).ti,kf. | 5903010 |
| 22 | or/19-21 | 9392287 |
| 23 | 18 not 22 | 462 |

**Supplemental Table S1b: Search in EMBASE**

| Database(s): Embase Classic+Embase 1947 to 2024 November 12 | |  |
| --- | --- | --- |
| Search Strategy: | |  |
| # | Searches | Results |
| 1 | exp heart infarction/ or exp acute coronary syndrome/ or exp ST segment elevation myocardial infarction/ | 546932 |
| 2 | ((cardi* or heart or myocardial*) adj3 (attack* or infarct* or failure* or arrest*)).ti,ab,kf. | 848319 |
| 3 | ((acute adj2 coronary adj2 syndrome) or AMICS or STEMI or NSTEMI or AMI).ti,ab,kf. | 134226 |
| 4 | (Left adj2 Ventricular adj2 Dominant adj2 Refractory).ti,ab,kf. | 1 |
| 5 | or/1-4 | 1057005 |
| 6 | exp cardiogenic shock/ | 43131 |
| 7 | ((cardi* or heart or myocardial* or cardiovascular or cardiocirculatory or circulatory) adj3 (shock* or collapse)).ti,ab,kf. | 49964 |
| 8 | (acute adj2 (heart or cardi* or myocardial* or cardiovascular or cardiocirculatory) adj2 failure).ti,ab,kf. | 27080 |
| 9 | or/6-8 | 88052 |
| 10 | (Impella* or (percutaneous adj2 ventricular adj2 assist adj2 device) or "pLVAD" or "pVAD" or "pMCS" or (microaxial adj2 flow adj2 pump)).ti,ab,kf. | 6634 |
| 11 | ((percutaneous or temporary) adj3 (circulat* or mechanical*) adj2 support*).ti,ab,kf. | 1956 |
| 12 | (percutaneous adj2 Mechanical* adj2 circulatory adj2 support).ti,ab,kf. | 351 |
| 13 | or/10-12 | 7879 |
| 14 | exp extracorporeal oxygenation/ | 50108 |
| 15 | (CardioHelp or RotaFlow or ECLS or ECMO or vaECLS or vaECMO or (venoarterial adj2 extracorporeal adj2 support)).ti,ab,kf. | 33330 |
| 16 | ((extracorporeal or "extra corporeal") adj2 (oxygen* or support)).ti,ab,kf. | 36107 |
| 17 | or/14-16 | 62457 |
| 18 | 5 and 9 and 13 and 17 | 1327 |
| 19 | exp case report/ or exp "review"/ | 6482454 |
| 20 | "case report".ti. | 441508 |
| 21 | ((exp animals/ or exp veterinary medicine/ or animal*.jw.) not exp humans/) or (experiment* model* or animal* or monkey* or sheep or ?ovine or lamb* or goat* or pig* or swine or porcine or pup* or dog* or canine or bitch* or beagle* or feline or rodent* or rabbit* or rat or rats or mouse or murine or mice).ti,kf. | 6904695 |
| 22 | or/19-21 | 13185923 |
| 23 | 18 not 22 | 789 |

**Supplemental Table S1c: Search in Cochrane Library**

| Search Name: | 240202 M Bogerd SR en ZonMW Impella vs ECMO cardiogenic shock |  |
| --- | --- | --- |
| Date Run: | 13-11-2024 17:35 |  |
| Comment: | |  |
|  |  |  |
| ID | Search | Hits |
| #1 | MeSH descriptor: [Myocardial Infarction] explode all trees | 15817 |
| #2 | MeSH descriptor: [Acute Coronary Syndrome] explode all trees | 3165 |
| #3 | MeSH descriptor: [ST Elevation Myocardial Infarction] explode all trees | 1168 |
| #4 | ((cardi* or heart or myocardial*) near/3 (attack* or infarct* or failure* or arrest*)):ti,ab,kw | 81368 |
| #5 | ((acute near/2 coronary near/2 syndrome) or AMICS or STEMI or NSTEMI or AMI):ti,ab,kw | 15275 |
| #6 | {or #1-#5} | 85280 |
| #7 | MeSH descriptor: [Shock, Cardiogenic] explode all trees | 497 |
| #8 | ((cardi* or heart or myocardial* or cardiovascular or cardiocirculatory or circulatory) near/3 (shock* or collapse)):ti,ab,kw | 2558 |
| #9 | (acute near/2 (heart or cardi* or myocardial* or cardiovascular or cardiocirculatory) near/2 failure):ti,ab,kw | 2875 |
| #10 | {or #7-#9} | 5335 |
| #11 | (Impella* or (percutaneous near/2 ventricular near/2 assist near/2 device) or "pLVAD" or "pVAD" or "pMCS" or (microaxial near/2 flow near/2 pump)):ti,ab,kw | 241 |
| #12 | ((percutaneous or temporary) near/3 (circulat* or mechanical*) near/2 support*):ti,ab,kw | 48 |
| #13 | (percutaneous near/2 Mechanical* near/2 circulatory near/2 support):ti,ab,kw | 14 |
| #14 | {or #11-#13} | 259 |
| #15 | MeSH descriptor: [Extracorporeal Membrane Oxygenation] explode all trees | 366 |
| #16 | (CardioHelp or RotaFlow or ECLS or ECMO or vaECLS or vaECMO or (venoarterial near/2 extracorporeal near/2 support)):ti,ab,kw | 1099 |
| #17 | ((extracorporeal or "extra corporeal") near/2 (oxygen* or support)):ti,ab,kw | 1294 |
| #18 | {or #15-#17} | 1718 |
| #19 | #6 AND #10 AND #14 AND #18 | 45 |

**Supplemental Table S2: Case Report Form**

| 1. **Study Characteristics** | | |
| --- | --- | --- |
| Title | ________________________________________________________ | |
| First Author | __________________ | |
| Year of publication | _ _ _ _ | |
| Link to full-text | Link: | |
| Country / Region |  | |
| Year of data query | Year data collection began: _ _ _ _, Year data collection ended_ _ _ _ | |
| Data Type | - Prospective - Post-Hoc Analyse - Retrospective; Chart Review - Retrospective; Claims database 🡪 _ _ _ _ _ _ _ _ _ _ _ _ _ _ - Retrospective; Propensity matched | |
| Inclusion / Exclusion | - Exclusion □ Inclusion, skip next question | |
| Reason for exclusion | - Irrelevant - Wrong population / AMI <50% - <10 cases per arm - Wrong Comparison - Wrong Outcome - Abstract Only - Review - Foreign Language - Duplicate | |
| Total number of AMICS patients | _ _ _ _ _ _ _ _ patients | |
| Percentage of AMICS patients | _ _ _ % | |
| Data formulated for AMICS separately | - No - Yes | |
| Number of patients with Impella primarily | _ _ _ _ _ _ _ _ patients | |
| Number of patients with VA-ECMO primarily | _ _ _ _ _ _ _ _ patients | |
| Definition used for stroke |  | |
| Definition used for bleeding |  | |
| Definition used for costs |  | |
| Definition used for discharge destination |  | |
| 1. **Data extraction per cohort (Impella and ECMO)** | | |
| **Baseline characteristics** | Impella cohort (n = _ _ _ _ _ _) | ECMO cohort (n = _ _ _ _ _ _) |
| Age, years | _ _ ±_ _ years | _ _ ±_ _ years |
| Male (%) | _ _ _ % | _ _ _ % |
| Diabetes Mellitus (%) | _ _ _ % | _ _ _ % |
| Lactate (mmol/L) | _ _ _ _ mmol/L | _ _ _ _ mmol/L |
| PCI (%) | _ _ _ % | _ _ _ % |
| CABG (%) | _ _ _ % | _ _ _ % |
| IABP (%) | _ _ _ % | _ _ _ % |
| **Outcomes** |  |  |
| In-hospital mortality | _ _ _ % | _ _ _ % |
| Length of ICU stay | _ _ _ _ _ _ _ _ days | _ _ _ _ _ _ _ _ days |
| Length of hospital stay | _ _ _ _ _ _ _ _ days | _ _ _ _ _ _ _ _ days |
| Costs (circle the currency) | _ _ _ _ _ _ _ _ € / $ | _ _ _ _ _ _ _ _ € / $ |
| Patients discharged home | _ _ _ _ _ patients | _ _ _ _ _ patients |
| Number of survivors | _ _ _ _ _ patients | _ _ _ _ _ patients |
| 1. **Risk of Bias using the Newcastle-Ottawa-scale (circle the stars you assign)** | | |
| **Selection** | Representativeness ★ non-exposed cohort ★ Exposure ★ Outcome at start ★ | |
| **Comparability** | Comparability of the cohorts: ★ ★ | |
| **Outcome** | Assessment ★, Follow-up length ★, Adequacy ★ | |
| **Total number of stars** | 1 2 3 4 5 6 7 8 9 ★ | |

**Supplements Table S3: Risk-of-Bias assessment according to the Newcastle-Ottawa Scale**

| Table S3. Risk of Bias assesses using the Newcastle-Ottawa Scale | | | | | | | | | |
| --- | --- | --- | --- | --- | --- | --- | --- | --- | --- |
|  | **Selection** | | | | **Comparability** | **Outcome** | | |  |
| First author | **Representative-ness of the exposed cohort** | **Selection of the non-exposed cohort** | **Ascertainment of exposure** | **Outcome not at start** | **Comparability (max 2 star)** | **Assessment of the outcome** | **Length of follow-up** | **Adequacy of follow-up of cohorts** | **Total Points (max 9)** |
| Maini | ★ | ★ | ★ | - | - | ★ | ★ | ★ | 6 x ★ |
| Karami | ★ | - | ★ | - | ★ | ★ | ★ | ★ | 6 x ★ |
| Lemor | ★ | ★ | ★ | - | - | ★ | ★ | ★ | 6 x ★ |
| Vallabhajo-syula | ★ | ★ | ★ | - | - | ★ | ★ | ★ | 6 x ★ |
| Pahuja (1) | ★ | ★ | ★ | - | - | ★ | ★ | ★ | 6 x ★ |
| Pahuja (2) | ★ | ★ | ★ | - | - | ★ | ★ | ★ | 6 x ★ |
| Vetrovec | ★ | ★ | ★ | - | ★ | ★ | ★ | ★ | 7 x ★ |
| Vojjini | ★ | ★ | ★ | - | - | ★ | ★ | ★ | 6 x ★ |
| Bogerd | ★ | ★ | ★ | - | ★ | ★ | ★ | ★ | 7 x ★ |
| Briasoulis | ★ | ★ | ★ | - | - | ★ | ★ | ★ | 6 x ★ |
| Padberg | ★ | ★ | ★ | - | ★ | ★ | ★ | ★ | 7 x ★ |
| Buda | ★ | ★ | ★ | - | - | ★ | ★ | ★ | 6 x ★ |
| Ali | ★ | ★ | ★ | - | ★ | ★ | ★ | ★ | 7 x ★ |

| First author (year of publication) | MCS | N | Age (years) | Male (%) | DM (%) | OHCA (%) | Lactate  (mmol/L) | pPCI (%) | CABG (%) | Hospital LOS (days) | Costs | % of survivors discharged home | In-hospital mortality (%) |
| --- | --- | --- | --- | --- | --- | --- | --- | --- | --- | --- | --- | --- | --- |
| Vallabhajos-yula (2020) | Incl.  IABP | 5248 | 63.5 ± 11.9 | 75 | - | 43 | - | 69 | - | 15.6 ± 20.1 | $555,000 ± $474,000 | 36 | 52 |
| Vojjini  (2021) | Incl.  IABP | 4816 | 63.2 ± 11.3 | 78 | - | 42 | - | 70 | - | 15.2 ± 20.0 | $508,000 ± $532,000 | 35 | 49 |
| Bogerd  (2023) | Excl. IABP | 429 | - | 80 | 17 | 38 | - | 100 | - | 17.6 ± 21.6 | €66,151 | 8 | 72 |
| Briasoulis (2023) | Incl.  IABP | 1494 | 58.6 (11.3) | 75 | 37 | 0 | - | - | - | 14.7 (18.5) | $153,996 ($128,935) | 15 | 58 |

**Supplemental Table S4: Data for cohort of ≥ 2 MCS devices**

**Supplemental Figures S5: Primary outcomes and in-hospital mortality including Briasoulis et al.**
Rationale for initial exclusion: The reported findings by Briasoulis significantly deviate from findings in other studies and preexisting medical knowledge. In addition, the studies by Buda, Ali and Briasoulis rely on data from a similar origin and timeframe (NRD- HCUP, 2016-2019/2020). Given this, the differences in the reported outcomes are difficult to understand.

S5a- length of hospital stay
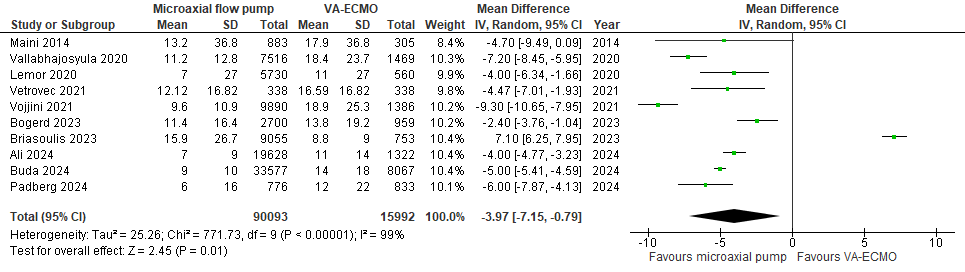
S5b - In-hospital costs
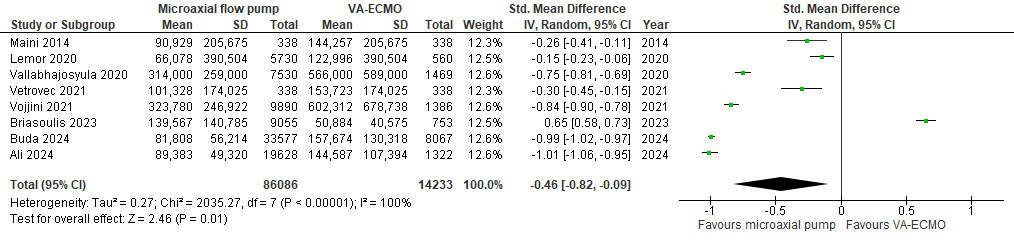


S5c – Discharge destination**
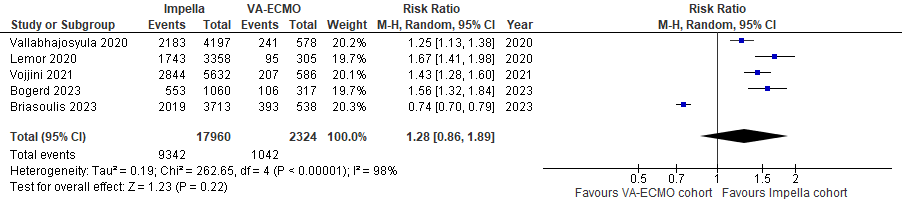
**

S5d - In-hospital mortality
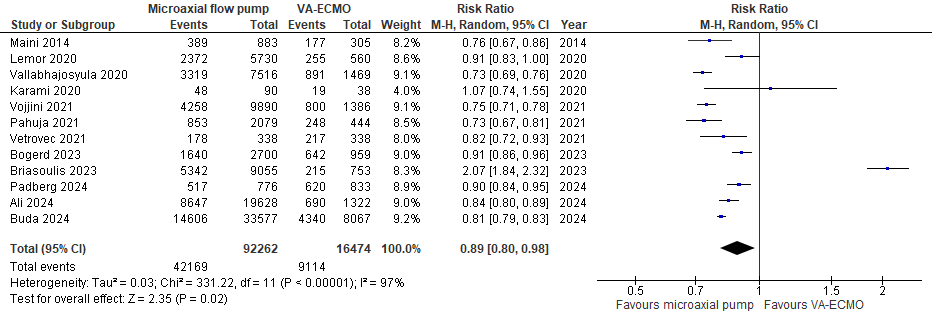


**Supplemental Figures S6: Meta-analysis of in-hospital mortality**


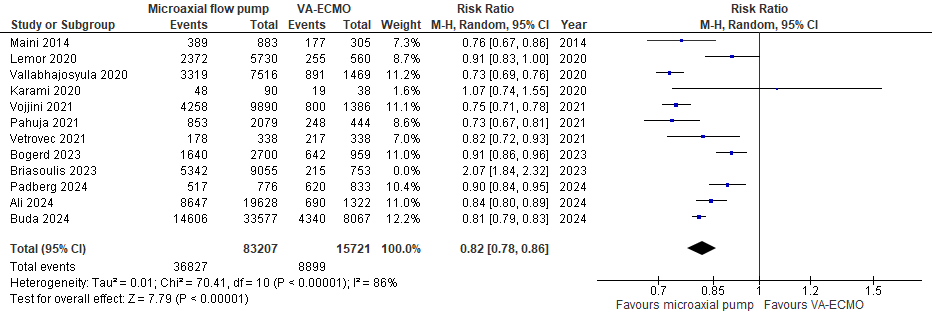


**Supplemental figures S7: Sensitivity analysis – Studies excluded to prevent overlap**Overlapping studies: Maini, Lemor, Vallabhajosyula, Pahuja, Vetrovec, Vojjini, Briasoulis, Buda, Ali.
Studies included: Karami, Bogerd, Padberg, Vallabhajosyula, Ali

S7a – in-hospital costs

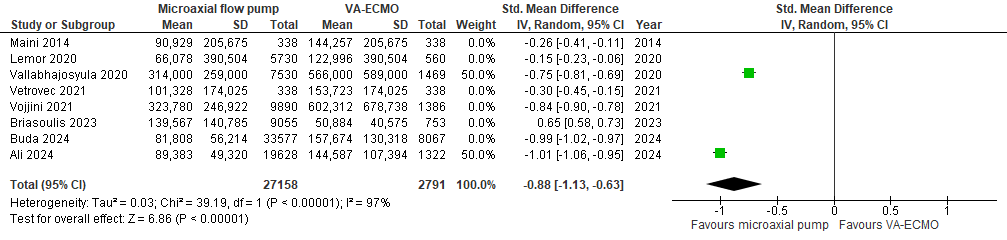


S7b – Length of hospital stay
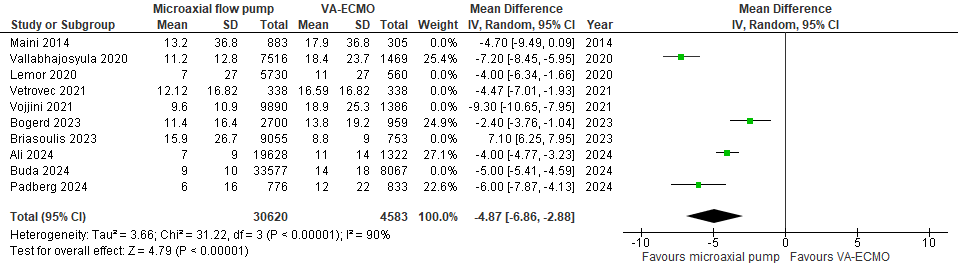


S7c – Discharge destination

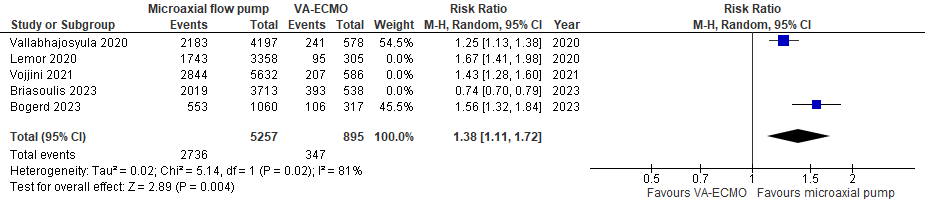


S7d. In-hospital mortality **
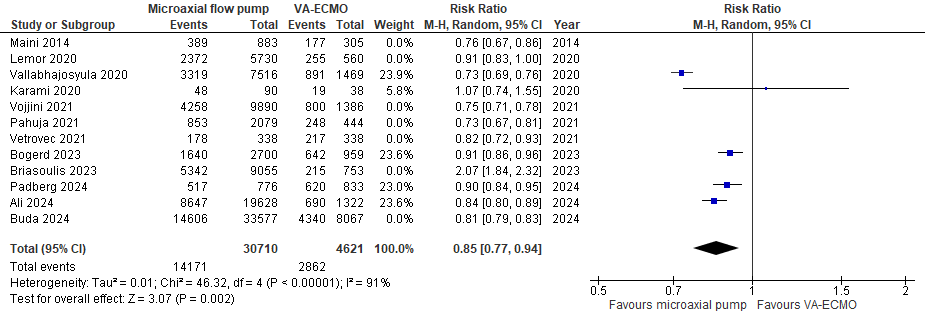
**

**Supplemental Figures S8: Sensitivity analysis – Studies from Europe vs. United States of America (USA)
Europe**: Karami, Bogerd, Padberg
**USA:** Maini, Lemor, Vallabhajosyula, Vojjini, Pahuja, Vetrovec, Briasoulis, Buda, Ali

S8a – Length of hospital stay in Europe
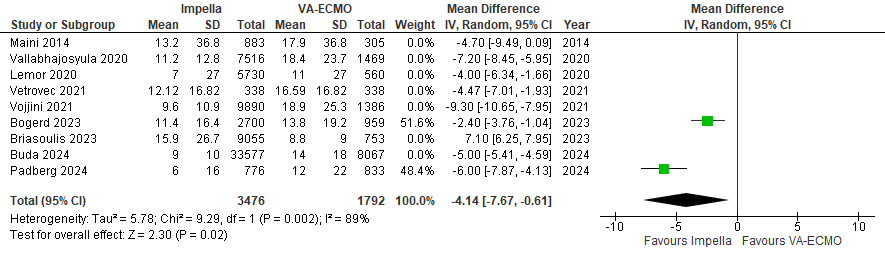
S8b – Length of hospital stay in the USA
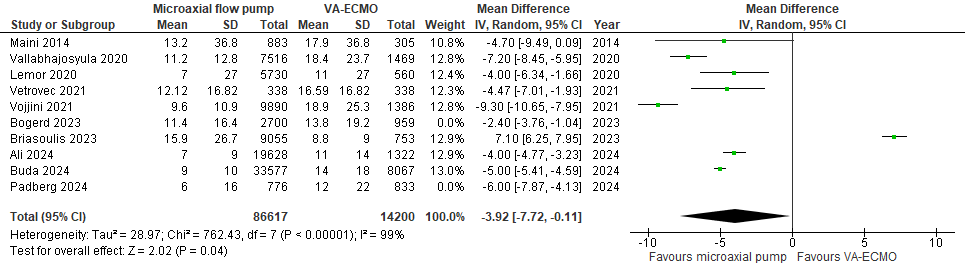


S8c – Discharge destination in Europe
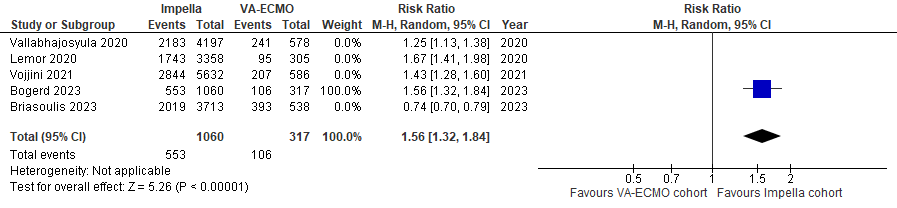
S8d – Discharge destination in the USA
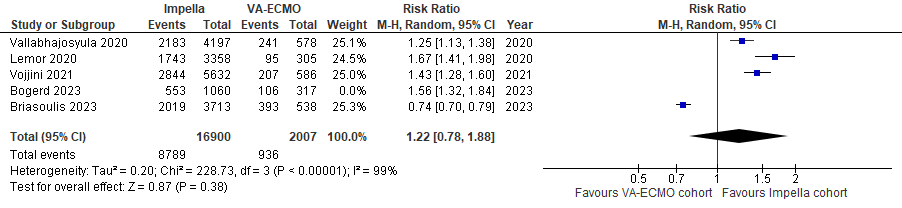


S8e. In-hospital mortality in Europe


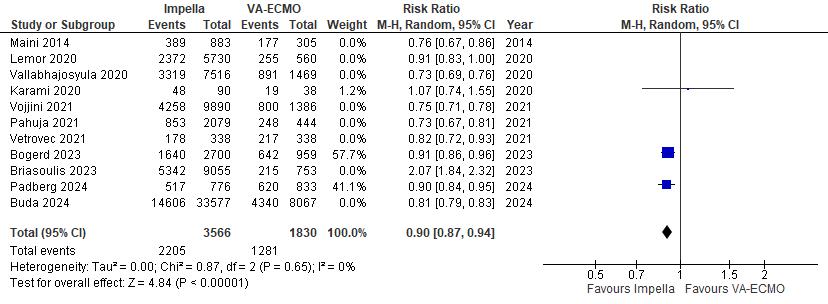
S8f. In-hospital mortality in the USA


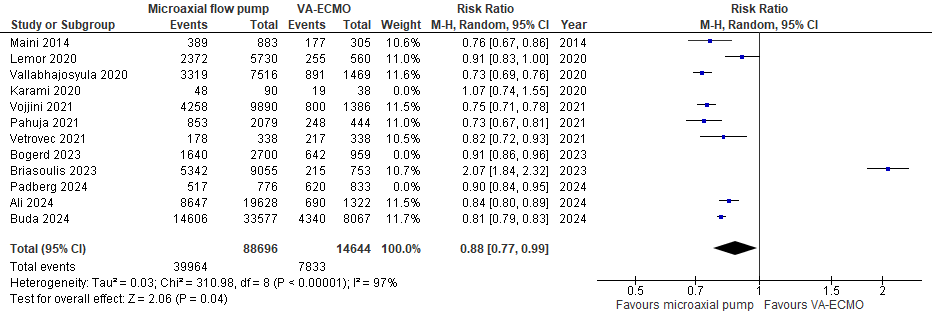


**Supplemental figures S9: Sensitivity analysis - Only studies with 100% PCI**Studies included: Karami, Lemor, Bogerd, Ali

S9a – in-hospital costs
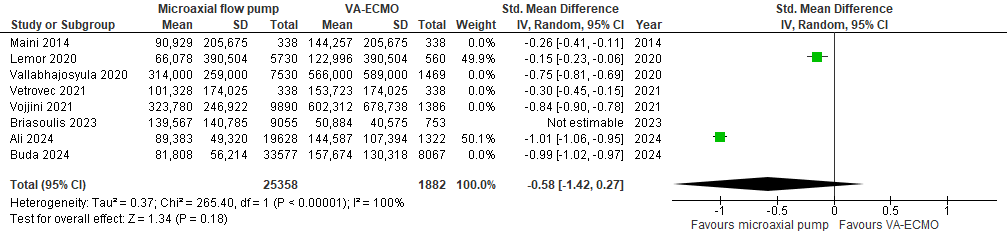


S9b – Length of hospital stay
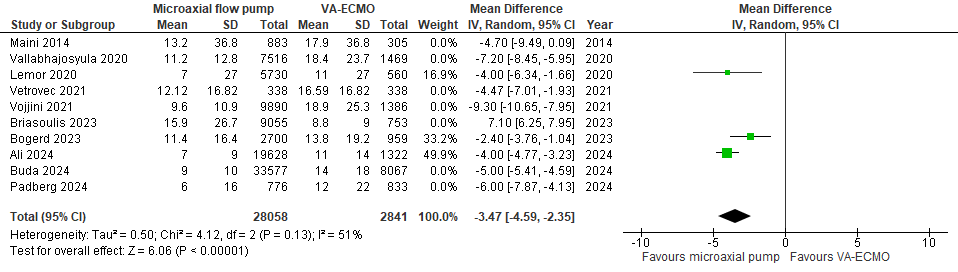


S9c – Discharge destination**
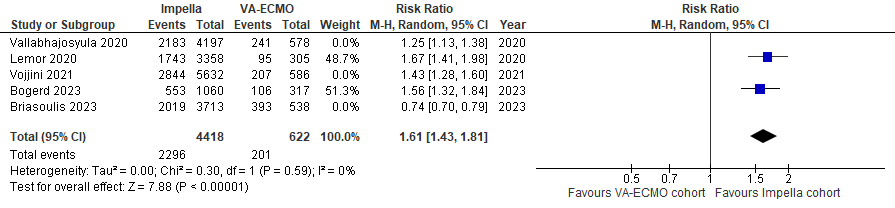
**

S9d. In-hospital mortality**
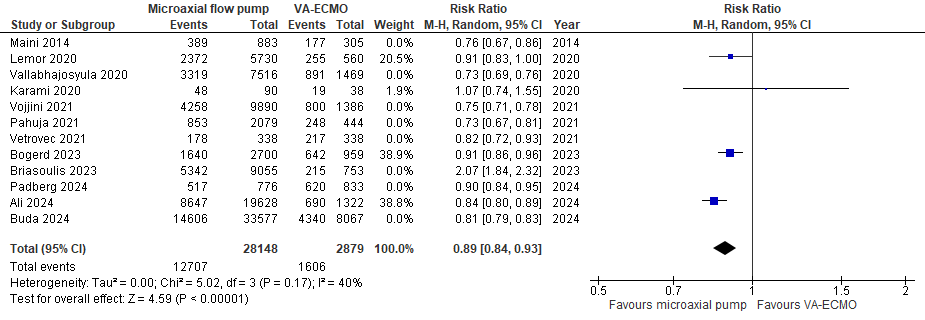
**

**Supplemental Figures S10- Fixed-effect models and funnelplots**

S10a – Length of hospital stay


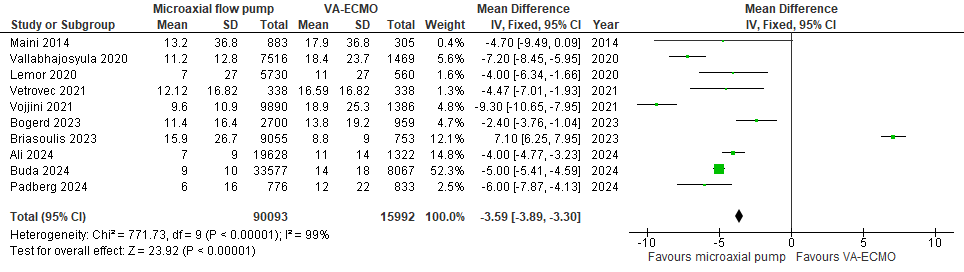


**
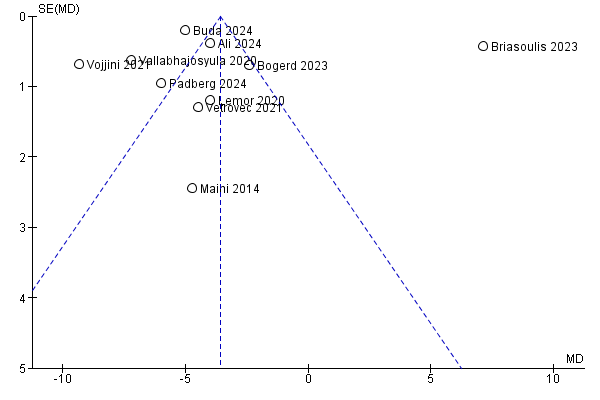
**

S10b- In-hospital costs

**
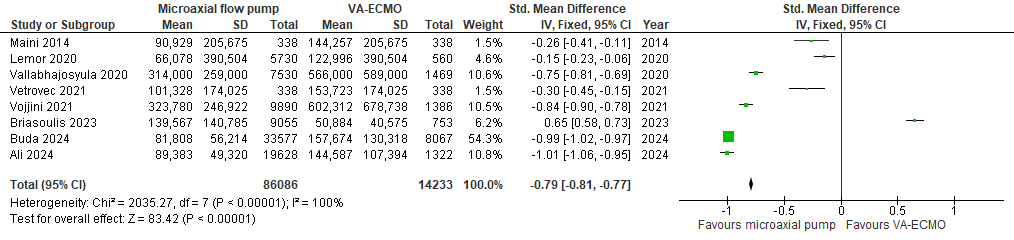
**


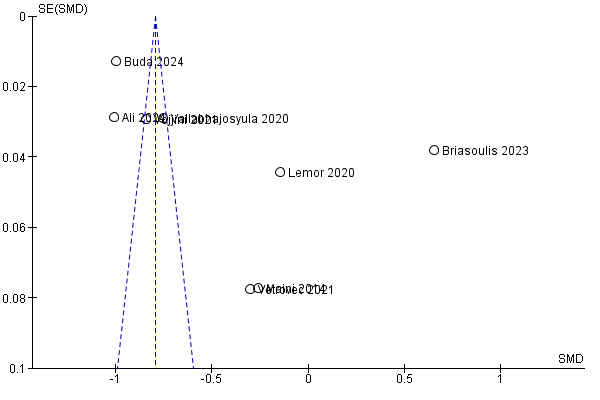


S10c - Discharge destination


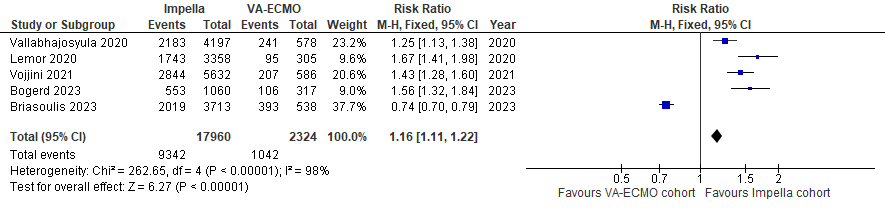


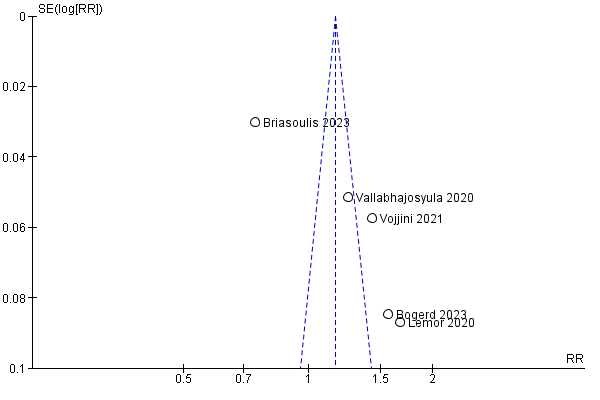


S10d - In-hospital mortality


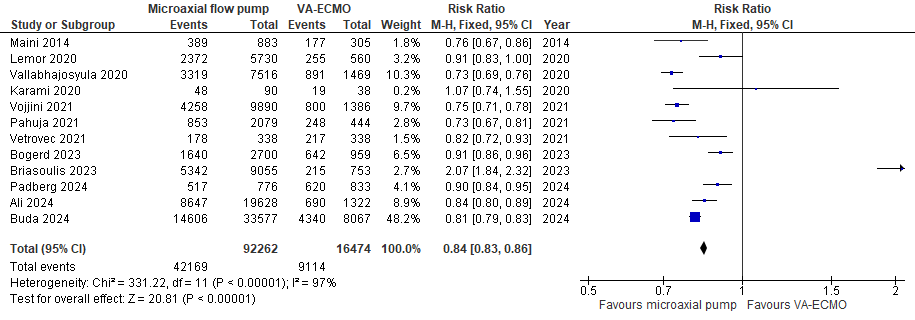


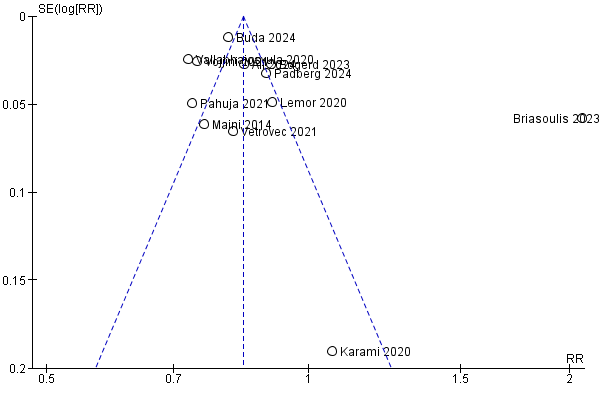

Supplement: zuaf024_Supplementary_Data [file zuaf024_supplementary_data.docx]
